# Predictors of graft patency following coronary artery bypass graft surgery: the role of nebivolol therapy

**DOI:** 10.1007/s11357-025-01688-5

**Published:** 2025-05-10

**Authors:** Tímea Balla, Tamás Maros, Gábor Csató, Nóra Erdei, Beatrix Ványai, Noel Johny Nellamkuzhi, Riko Shima, Dániel Czuriga, Zoltán Csanádi, Andrea Molnár, Nóra Homoródi, Zsolt Kőszegi, Attila Kiss, István Édes, Gábor Tamás Szabó

**Affiliations:** 1 Department of Cardiology and Cardiac Surgery, Faculty of Medicine, University of Debrecen, Debrecen, Hungary; 2 Department of Dermatology, Faculty of Medicine, University of Debrecen, Debrecen, Hungary; 3 Center for Biomedical Research and Translational Surgery, Medical University of Vienna, Vienna, Austria

**Keywords:** Coronary artery bypass graft surgery, Graft patency, Grafts degeneration, Nebivolol

## Abstract

The long-term postoperative occlusion of venous or arterial grafts following coronary artery bypass graft surgery (CABG) is a constant and unresolved problem, with a negative impact on clinical outcome. In our study, we aimed to find predictors that influence graft patency. The data of 202 patients who underwent CABG and had control coronary angiography on an average of 8.55 ± 4.56 years were analyzed retrospectively. Based on the presence of graft occlusion ascertained at control coronary angiography, patients were divided into two groups: 81 in the graft occlusion group (with 89 grafts degeneration: 64 saphenous vein [SVG] and 25 arterial graft) and 121 in the control group with patent grafts. The two groups were considerably well-matched regarding patient characteristics. Among medical conditions, peripheral artery disease has been found to be an independent predictor of increased graft occlusion, but only in SVG patients (OR 3.64, CI 1.21–11.03, *p* = 0.021). When evaluating medical therapy, significantly more patients were on nebivolol in the control group, compared to the graft occlusion group (*p* = 0.032). Moreover, nebivolol has been found to be an independent predictor of a lower degree of graft occlusion development (OR 0.36, CI 0.14–0.94, *p* = 0.036). Nebivolol has been found especially effective in preventing SVG graft occlusion (OR 0.24, CI 0.07–0.76, *p* = 0.015). Nebivolol has been found to reduce the frequency of graft occlusion following CABG, especially in case of SVG grafts. The vasodilatory properties of nebivolol may, at least in part, be responsible for the favorable effects of the drug to prevent graft occlusion.

## Introduction

Cardiovascular (CV) diseases are still the leading cause of mortality worldwide [1]. To prevent acute coronary syndrome (ACS) and to improve symptoms and prognosis in chronic coronary syndrome (CCS), coronary artery revascularization by coronary artery bypass grafts (CABG) is a frequently used therapeutic option. In patients, being considered for coronary revascularization, current guidelines [2, 3] emphasize the multidisciplinary Heart Team approach to optimize patient selection for CABG. In suitable patients CABG is suggested for primary revascularization in cases with (1) left main stem stenosis, and (2) complex diabetic multivessel coronary disease [2, 3] to improve symptoms, reduce mortality, and repeat revascularizations. However, even in suitable patients, the long-term postoperative occlusion of surgical grafts following CABG is a constant and unresolved problem. After CABG, different drugs are used [2] to preserve graft patency (lipid-lowering drugs, antiplatelets therapy, and calcium channel blockers in case of radial artery grafting).

Following CABG, the benefits of beta blockers (BB) therapy have been clearly established in patients with previous acute infarction, part of the secondary CV prevention or with left ventricular dysfunction [2]. Moreover, the BBs a widely used following CABG to prevent the new onset of atrial fibrillation [2].

The aim of our study was to find novel patient- and CV therapy-related independent predictors, that could potentially affect graft patency. In our data analysis, special focus was given to the BB treatment, since BB therapy improves antianginal symptoms in different ways, including the vasodilatory effects such as in case of nebivolol treatment.

To identify factors that may influence graft patency, we evaluated our CABG database in a retrospective study with focus on (1) CV pharmacotherapy; (2) demographic factors, comorbidities, and CV risk factors. Our main objective was to examine the difference between patients with and without graft occlusion with the expectation that the optimal pharmacotherapy may lead to a higher degree of graft patency.

## Methods

### Study population

Data collection was carried out on all consecutive patients who underwent coronary angiography for any indication in our centre between June 1, 2015, and May 31, 2016, and had a history of CABG. The indication for CABG was multivessel CCS. The indication for the control angiography was acute coronary syndrome (ACS), crescendo angina, new onset of angina (CCS), or diagnostic coronary angiography due to any other cardiac conditions (such as valvular diseases with surgical- and interventional indications). ACS was diagnosed when the criteria of the universal definition of myocardial infarction were met [4–6]. The intervention and the medical therapy of patients included in this study were in line with the guidelines in effect on coronary revascularization. Pre-hospital triage with transtelephonic ECG and direct referral for catheter-based therapy were used, for patients with the diagnosis of ACS [7, 8].

### Parameters evaluated in the study

Medical records were collected to analyze demographic parameters including age and sex, and comorbidities (hypertension, diabetes mellitus, peripheral arterial disease, and chronic renal impairment), CV pharmacotherapy, resting heart rate, and echocardiography. Resting heart rate was calculated from the ECG registrations at the time of coronary angiography. Examination of the qualitative blood counts was done at the time of cardiac surgery. The focus was on the CV inflammation marker neutrophil to lymphocyte ratio (NLR) [9].

The results of transthoracic echocardiography (left ventricular ejection fraction and left ventricular wall motion abnormalities) were evaluated at the time of control coronary angiography. To calculate ejection fraction the Simpson’s formula of the software package was used (Acuson-Sequoia device with a 3.5 MHz harmonic imaging transducer).

Coronary anatomy data were carefully analyzed, and all graft obstructions (no flow phenomenon) were identified by two independent invasive cardiologists.

The study was conducted in accordance with the Declaration of Helsinki. Study data was collected with the written consent of the patients. Data management and collection procedures were approved by the institutional review boards of the Department of Cardiology and the University of Debrecen, Hungary (Protocol code: 5903–2021).

### Statistical analysis

A statistical analysis was performed by the GB-Stat v8.0 program (Dynamic Microsystems Inc.). Normally distributed continuous variables were compared by using Student’s *t* test, and the Wilcoxon rank-sum test was used to compare the analysis of categorical variables (in both cases *α*-level of 5%).

Predetermined variables between patients in the graft occlusion and control groups with a *p* < 0.6 were assessed by applying a univariate logistic regression model. Odds ratios (OR) and 95% confidence intervals (CI) were calculated. In case of multiple regression, predictors of graft occlusion displaying a *p* value of < 0.15 in a univariate analysis were selected and quantified for adjusted odds ratios and CIs. A *p* value of < 0.05 was regarded as significant.

## Results

Altogether, 202 patients who underwent CABG with the indication of multivessel CCS were involved in the study. The mean age of the patients was 58.81 ± 8.58 years. During cardiac surgery, saphenous vein grafts (SVG), left internal mammary artery (LIMA) grafts, right internal mammary artery (RIMA) grafts, and radial artery grafts were used. The implanted graft/subject number was 2.2. In our patient’s cohort (Table 1), after 8.55 ± 4.56 years following CABG, altogether 89 graft occlusions were noted in 81 patients, (40.09%) by control coronary angiography. The indications for follow-up coronary angiography were ACS, new onset angina, or any other cardiac condition requiring invasive clarification of the coronary status. Proportionally, the graft degeneration was significantly higher for the SVG (36.2%), compared to the arterial grafts (13.7%).

**Table 1:** Number of patients in the different populations

	Total population	Graft occlusion population
Total number of patients with grafts (***n***)	202	81
° Number of patients with arterial graft	175	24
Number of patients with LIMA graft	170	13
Number of patients with RIMA graft	6	2
Number of patients with radial artery graft	25	10
Number of patients with SVG graft	177	64

Values are number of patients. In some cases, more than one graft was occluded. The average implanted graft/patient ratio was 2.2 in the total patient population (*n* = 202). *CABG*, coronary artery bypass graft; *LIMA*, left internal mammary artery; *RIMA*, right internal mammary artery; *SVG*, saphenous vein graft

Based on the presence of graft occlusion, study participants were divided into two groups: 81 patients in the graft occlusion group and 121 patients in the control group with patent grafts. The baseline characteristics and the cardiovascular (CV) pharmacotherapy at the time of coronary angiography are presented in Table 2*.* Slightly more previous ACS and peripheral artery disease (PAD) were noted in the occlusion group compared to the control; however, the differences were not statistically significant. The two groups were relatively well-balanced regarding CV risk factors and other concomitant diseases. No significant difference was noted in the CV inflammation marker NLR between groups. The graft occlusion group had a slightly significantly (*p* = 0.013) higher basal heart rate, compared to controls.

**Table 2:** Baseline clinical characteristics, selected laboratory parameters, and medical therapy

Parameter	Total patient population (*n* = 202)	Control—patients with patent grafts (*n* = 121)	Patients with graft occlusion (*n* = 81)	*p* value
Age (y)	58.81 ± 8.58	59.33 ± 8.42	58.02 ± 8.81	0.294
Male gender (%)	74.26	74.38	74.07	0.971
Female gender (%)	25.74	25.62	25.93	
Time after CABG (years)	8.55 ± 4.56	8.15 ± 4.37	9.16 ± 4.79	0.130
Basal frequency (beat/min)	80.26 ± 9.34	78.89 ± 9.04	82.31 ± 9.47	0.013
Hypertension (%)	77.72	82.64	70.37	0.139
Diabetes (%)	31.68	32.23	30.86	0.869
PAD (%)	8.42	5.8	12.34	0.429
Renal failure (%)	2.97	1.10	4.94	0.692
Left ventricular EF (%)	49.06 ± 7.72	48.78 ± 7.83	49.54 ± 7.57	0.562
Heart failure (%)	23.17	20.89	26.98	0.532
Previous ACS (%)	34.75	30.69	41.27	0.283
Qualitative blood counts				
Neutrophils (%)	65.65 ± 9.42	65.00 ± 10.05	66.61 ± 8.40	0.237
Lymphocytes (%)	24.64 ± 8.68	25.01 ± 8.78	24.25 ± 8.18	0.548
NLR	3.31 ± 2.46	3.30 ± 2.78	3.31 ± 1.12	0.929
Pharmacotherapy				
Statin	71.95	70.29	74.60	0.643
ASA and/or clopidogrel	95.15	95.04	95.31	0.991
ASA	89.63	91.08	85.71	0.577
Clopidogrel	35.36	35.64	34.72	0.988
Trimetazidine (%)	65.84	66.949	64.19	0.766
ACEi and/or ARB (%)	85.64	82.64	90.12	0.368
ACEi (%)	80.69	76.86	86.12	0.249
ARB (%)	12.87	13.22	12.34	0.957
BB (%)	96.53	95.87	97.53	0.393
Nebivolol (%)	15.84	20.66	8.64	0.032
Nitrate (%)	43.07	44.17	41.96	0.792

Values are means ± SD or percentages of subjects. *ACE*, angiotensin converting enzyme inhibitor; *ACS*, acute coronary syndrome; *ARB*, angiotensin II receptor blocker; *ASA*, acetyl salicylic acid; *BB*, β-receptor blocker; *CABG*, coronary artery bypass graft; *EF*, ejection fraction; *HF*, heart failure; *NLR*, neutrophil/lymphocyte ratio; *PAD*, peripheral artery disease. In this instance, the *p* value refers to differences between patient groups with and without graft occlusion

Standard care of CV pharmacotherapy was used in the study population according to guidelines in effect (Table 2). In most of the patients, the lipid-lowering drugs, antiplatelets therapy, angiotensin-converting enzyme, inhibitors/angiotensin II receptor blockers, and BBs (including nebivolol) were already initiated before CABG. In the postoperative period, nebivolol was given in an average daily dose of 6.4 mg. Daily doses and the adherence to pharmacotherapy in the postoperative period were self-reported by the patients at the time of control coronary angiography.

Among the two groups, there was no significant difference in CV pharmacotherapy with one exception: significantly (*p* = 0.032) more patients were on vasodilatory beta-blocker therapy (nebivolol) in the control group, compared to the graft occlusion group (Table 2). For the other BBs (bisoprolol and metoprolol), no difference was observed between the groups. Interestingly, we could not detect significant effect on graft occlusion with other vasodilators (nitrate treatment). In fact, the administration of nitrates only slightly, but not significantly reduced the graft occlusion rate (Table 2).

**Fig. 1:**
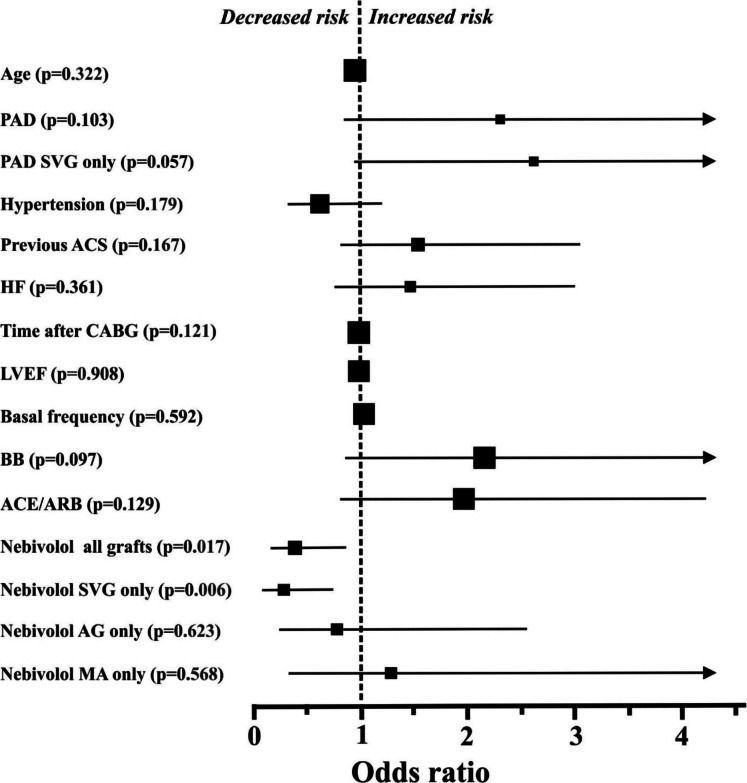
Odds ratios and 95% confidence intervals for graft occlusion in the individual subgroups, defined based on baseline characteristics and drug therapy. The sizes of the symbols reflect the number of patients in each group. For some parameters (age, frequency, time after CABG, and LVEF), confidence intervals are within the symbols. ACE/ARB, ngiotensin converting enzyme inhibitors/angiotensin II receptor blockers; ACS, acute coronary syndrome; AG, arterial graft; ASA, acetyl salicylic acid; BB, beta blockers in total; CABG, coronary artery bypass grafts; HF, heart failure; LVEF, left ventricle ejection fraction; MA, internal mammary arteries; PAD, peripheral artery disease; SVG, saphenous venous grafts

All predetermined parameters between the two groups with *p* < 0.6 were evaluated by the univariate log-rank test (Fig. 1). The ORs and CIs for graft occlusion were calculated. Applying the univariate statistical method, only nebivolol treatment turned out to exert significant effects on the development of graft occlusion. Nebivolol was especially effective on SVG occlusion. On the other hand, in the case of arterial grafts (LIMA, RIMA, and radial artery), nebivolol treatment was without significant effect on patency (Fig. 1). If the internal mammary grafts were examined only, similar results were noted (Fig. 1). Interestingly, in case of the limited number of radial artery occlusions (10 cases), no patients were on nebivolol. Our data analysis showed that PAD and previous ACS increased the risk for occlusion; however, in the univariate analysis, these changes were statistically not significant (*p* = 0.103 and 0.167, respectively).

In univariate analysis, variables with a *p* < 0.15 were selected for multiple regression and were quantified for adjusted ORs and CIs for graft occlusion. Nebivolol therapy (OR 0.36, CI 0.14–0.94, *p* = 0.036) was a strong independent predictor that decreased the risk of graft occlusion in our model. When the analysis was done on the SVG patients only, the risk reduction was even more significant (OR 0.24, CI 0.07–0.76, *p* = 0.015). At the same time, PAD also proved to be an independent predictor in SVG patients and increased the risk of graft occlusion (OR 3.64, CI 1.21–11.03, *p* = 0.021). All other parameters below *p* < 0.15 in the univariate analysis, were not found to be an independent predictors of the graft occlusion in our patient population, (i.e., ACE/ARB, BB treatment in total and time after CABG).

## Discussion

The incidence of SVG occlusion varies widely in different studies and registries. In different databases, the 1-, 5-, and 10-year postoperative patency rates are 93%, 74%, and 41%, respectively [10, 11]. In addition, some studies have shown that up to 10–15% of SVGs may become occluded within the first 6 months after CABG [12]. In our study population, the SVG patency rate was found in similar range (64%) 8.55 ± 4.56 years after CABG. In the development of SVG occlusion, various pathogenic mechanisms have been previously proposed. These are the (1) early thrombus formation (< 1 month after CABG), (2) neointimal hyperplasia (1–12 months after CABG), and (3) atherosclerosis (> 1 year after CABG) [13]. Regarding chronic atherosclerosis as a risk factor, in the present study, we also noted that PAD is associated with increased SVG occlusion.

Many factors have been reported to increase the risk of SVG occlusion, such as surgery-related factors [14], smoking [15], diabetes mellitus [15], hyperlipidemia [16], and other blood chemistry changes [17]. Applying secondary preventive medical therapy (lipid-lowering and antidiabetic drugs), adequate patients’ education (smoking cessation) and improved surgical techniques most of these risk factors can be addressed. Lipid-lowering therapy reduces the lipid rich plaque formation and intimal hyperplasia (smooth muscle cell proliferation) in the grafts [18]. The target low-density cholesterol level following CABG has been suggested to be below 2.5 mmol/l [18].

In addition, recent guidelines [2, 3] emphasize the early administration of low dose of aspirin following CABG (class IA recommendation) to improve graft patency and reduce early thrombus formation and ischemic complications. In selected high-risk patients, even the double anti-platelet therapy (aspirin and ticagrelor or clopidogrel) has been suggested for 1 year to improve graft patency [2, 19]. However, further studies and data are needed to establish the safety, efficacy, and the exact duration of double anti-platelet therapy to prevent graft occlusion. In accordance with the above recommendations, most of our patients (> 95%) were on aspirin and/or clopidogrel following CABG (Table 2). Consequently, based on our database, the effect of these drugs on graft patency could be addressed only with limited power.

In our study, significant beneficial effects of nebivolol treatment have been suggested on the prevention of graft occlusion development. Nebivolol was especially effective in protecting SVG patency (Fig. 1). In general, no effect of nebivolol was observed on the patency of arterial grafts (LIMA, RIMA, and radial artery). However, in the limited number of radial artery occlusions (10 cases), no patients were on nebivolol. This observation is interesting and may indicate that the vasodilatory effect of nebivolol varies between arterial grafts (the effect is not homogeneous). The mechanism of graft occlusion has been reported to be different between arterial grafts and SVGs. Acute thrombosis and late atherosclerosis are observed predominantly in SVGs, and patients with radial artery grafts commonly develop vasospasm [20]. Consequently, both the recent European Task Force consensus opinion [20] and the ACC/AHA/SCAI Guideline for Coronary Artery Revascularization [2] propose oral calcium channel blocker (amlodipine or diltiazem) administration for 1 year post-operatively after radial artery grafting (vasodilatory treatment). The patients enrolled in our database had CABG operation in the years 2008–2013, consequently no calcium channel blocker was used postoperatively (the recommendation was still not available).

Nebivolol is regarded as a highly beta1-selective blocker and widely used in clinical practice [21, 22]. Depending on the experimental conditions, the selectivity ratio (ratio of beta1- to beta2-blockade) of the drug has been reported to be between 4 and 46 in human ventricular myocardium [23, 24]. Moreover, clinical studies have demonstrated that nebivolol possesses an additional nitric oxide (NO)-mediated vasodilatory effect which was not observed for other beta-blockers (e.g., atenolol and metoprolol) [22]. Previous data indicate [25] that nebivolol (administered preoperatively before CABG) significantly increases the NO levels in both SVG and arterial grafts at the vascular graft endothelium and vasa vasorum. The higher NO levels in the graft of patients undergoing CABG makes nebivolol safer and superior compared to other beta blockers to preserve vascular endothelial function (graft relaxation) and prevent vasospasm [25] and myocardial hypoperfusion [26]. It has been suggested that the careful handling of SVG grafts (using proper storage and rinsing solutions) during cardiac surgery is an important factor in preserving the human endothelial cell function [27].

Various mechanisms have been suggested to be responsible for the NO-mediated vasodilatory effect of nebivolol, but the direct effects on the endothelial continuous NO release (L-arginine/NO pathway) seem to be the most important [28, 29]. The nebivolol-induced improved endothelial function and increased NO production may be the key factors to preserve patency of a vein when grafted into an arterial environment. Moreover, it also explains why nitrate administration may not be as effective as nebivolol treatment. To avoid organic nitrate tolerance (loss of efficacy), nitrates are administered intermittently (night hours are nitrate-free). On the other hand, nebivolol maintains a 24-h continuous NO production. Regarding the preservation of vascular endothelial function, theoretically, the continuous NO production may be superior.

In a previous study [30], a significant beneficial effect of the anti-inflammatory drug trimetazidine has been demonstrated on the development of in-stent restenosis (ISR) following PCI. On the other hand, in our database, trimetazidine did not show any effect on graft patency following CABG. It should be emphasized however, that: (1) in the development of graft occlusion and ISR, different pathogenic mechanisms have been proposed, and (2) trimetazidine therapy after PCI was effective only in a subgroup of patients (bare-metal stent implantation and narrow coronary arteries).

Since, the inflammation is regarded as an important determinant for both the initiation and progression (intimal hyperplasia) of coronary artery disease [31], the relationship between anti-inflammatory treatment and graft occlusion should be clarified in future clinical studies.

## Conclusions

The main aim in this study was to find new independent predictors, which may influence the development of graft occlusion, in CABG patients. In accordance with previous studies, graft occlusion was significantly more frequent for SVG compared to arterial grafts (LIMA, RIMA, and radial artery). In our patient population, nebivolol treatment had a significant beneficial effect on graft patency and was found to be an independent predictor for decreasing the risk of graft occlusion. The vasodilatory properties of nebivolol to protect graft occlusion have been found to be especially useful on SVG patients.

## Limitations

All presented data are from one cardiac center, and the data analyses were conducted on a retrospective basis. This creates a potential limitation for our study. Another limitation of our study is that some relevant variables of graft occlusion were not examined (due to the relatively low patient number) and incorporated into our model (e.g., surgical technique variations and differences in acute postoperative care). Moreover, the retrospective database analysis carries the risk of some selection bias. Patients with symptoms of newly developed angina could have been referred more frequently for follow-up angiography compared to other CABG patients.

However, it should be emphasized that: (1) the data collection period was relatively long, (2) all CABG patients with follow-up angiography were included in the database, and (3) the SVG patency rate of our study population was found to be similar for those reported by previous publications. Moreover, the baseline characteristics of the examined groups (graft occlusion and control) were considerably well-matched.

Overall, we think that the finding (i.e., nebivolol may reduce SVG graft occlusion) is important and requires further studies using a multicenter approach and larger database. Moreover, the graft occlusion protective effect of nebivolol should also be examined on radial artery grafts.

## Data Availability

The data that support the findings of this study are available from the corresponding author upon reasonable request.
